# Analytical tests to evaluate pozzolanic reaction in lime stabilized soils

**DOI:** 10.1016/j.mex.2020.100928

**Published:** 2020-05-28

**Authors:** Pavan Akula, Dallas N Little

**Affiliations:** a Research Assistant, Texas A&M University; b Regents Professor, Texas A&M University

**Keywords:** Calcium Silicate Hydrate, Chemical modification, Lime stabilization, Shrink-swell soils, Analytical testing, X-Ray diffraction, Differential Thermogravimetric analysis, Pozzolanic reaction

## Abstract

Shrink-swell soils are predominant in various parts of the parts of the world. Lime has been extensively used to reduce the shrink-swell mechanism as it chemically reacts with soil minerals forming pozzolanic products such as calcite and calcium-silicate-hydrate (C-S-H). Conventionally, whether chemical treatment of soils results in effective pozzolanic stabilization reactions is determined anecdotally through engineering tests including unconfined compressive strength, plasticity index (PI), and pH tests. This study builds on existing literature regarding how more direct quantification of pozzolanic products can be obtained through tests that directly identify and quantify pozzolanic products, specifically in lime-treated clay soils. Specifically, x-ray diffraction (XRD) and differential thermogravimetric analysis (DTA) are used for this testing. Expansive soils with plasticity indices above 25% were selected for this study. Engineering tests on these lime-treated soils indicated significant improvement in strength and reduction in PI. In XRD analysis, pozzolanic products are assessed by the location and intensity of x-ray peak(s). The XRD data show a decrease in the intensity of alumio-silicate minerals such as kaolinite and smectite; silica and alumina are dissolved at a high pH and converted to pozzolanic products such as calcium-silicate-hydrate (C-S-H). DTA indicates the presence of C-S-H with the characteristic weight loss from 140°C to 250°C.The methodology describes the following:

● Sample preparation steps for XRD and DTA analysis.

● Analysis of XRD results and DTA analysis.

## Graphical abstract

**Figure:**
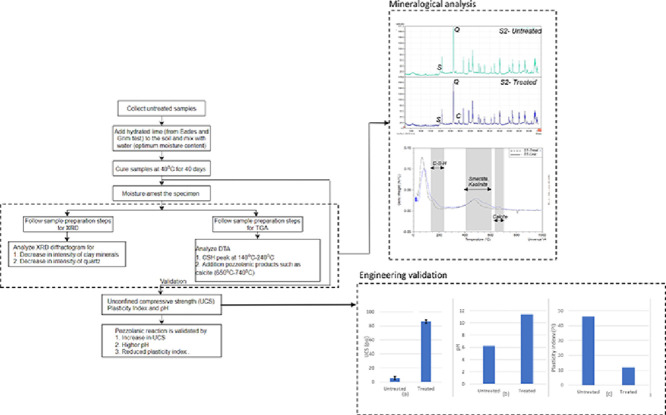

Specifications Table

**Table:** 

Subject Area	Engineering
More specific subject area	Method to evaluate pozzolanic reaction in lime stabilized soils
Method name	Analytical tests for C-S-H identification in lime treated soils
Name and reference of original method	N/A
Resource availability	N/A

## Introduction

Expansive clay minerals are prevalent in many parts of the western and southwestern U.S. Soils containing such expansive minerals are referred to as shrink-swell soils due to their swelling characteristics in the presence of moisture and shrinking characteristics during moisture loss. The amount of actual swell is dependent on the moisture condition, climatic conditions, and the type of expansive clay mineral present. In the U.S. alone the annual cost due to damage caused by expansive soils is estimated to be $2.3 billion [1]. Montmorillonite, a smectite group clay mineral, is the most prominent expansive clay mineral. Calcium based chemical additivities including lime, cement, and other cementitious materials are effective in stabilizing expansive soils.

Several researchers have studied the change in physical and engineering properties for lime-treated soils [2], [3], [4], [5], [6], [7], [8]. Little [4] explained that addition of lime to reactive soils substantially increases the resilient modulus (by a factor of 10 or more) and strength (by a factor of 20 or more in some cases). Bell [9] determined that addition of a small percentage of lime to soil enhanced the engineering properties and improved constructability. In addition to improvement of physical and engineering properties, studies have identified mineralogical and geochemical changes in lime-treated soils [10], [11], [12]. Microstructural investigations have reported the presence of calcium-silicate-hydrates and calcium-aluminate-hydrates in lime treated soils [10,[13], [14], [15], which bind the soil particles together in a strong matrix. Investigations [14], [15], [16], [17], [18] have shown that soils treated with supplementary cementitious materials such as fly-ash and cement-kiln dust produce similar strength enhancing pozzolanic products.

There is a deficit of literature that provides step-by-step instructions on how to prepare and test samples in order to perform x-ray diffraction (XRD) and differential thermogravimteric analysis (DTA). For example, there is a lack of information on the recommended instrumentation parameters and analysis steps for XRD and DTA. This study addresses those shortcomings by building on existing literature and providing a detailed step-by-step approach that can be readily followed by practicing engineers on field collected soil samples. This study emphasizes sample preparation and analysis methodology for XRD and DTA. The objectives of the study are:

1. Describe detailed sample preparation steps for analytical tests such as XRD and DTA.
2. Detail steps to analyse XRD and DTA results.
3. Compare XRD and DTA results with conventional engineering test results such as unconfined compressive strength (UCS) and Plasticity Index (PI).

### Stabilization process

Chemical stabilization of expansive soils with additives such as lime, portland cement (cement) and fly ash has been successfully achieved as evinced by strength increase and reduction in shrink-swell behavior upon treatment [19], [20], [43], [44]. Addition of hydrated lime (Ca(OH)_2_) improves the compressive strength of the stabilized material [21]. When hydrated lime is added to the soil, it disassociates into Ca^2+^ and OH^−^ ions. The release of OH^−^ ions increases the pH to 12.4, which causes silica and alumina from clay minerals to dissolve, and in combination with Ca^2+^ ions form calcium-silicate-hydrates (C-S-H) and calcium-aluminate-hydrates (C-A-H). The increase in strength is largely attributed to the formation of these C-S-H and C-A-H products, an amorphous gel that binds the soil matrix. The quantity of C-S-H and C-A H that can form at chemical equilibrium is dependent on the soil mineralogy, pH and percentage of Ca(OH)_2_ added. A simplified qualitative view of typical soil-lime reactions [22], [23], [24] are as follows:

$$\text{Ca}{\left(\text{OH}\right)}_{2}\to Ca^{2+}+2{\left(\text{OH}\right)}^{-}$$

$$Ca^{2+}+2{\left(\text{OH}\right)}^{-}+\text{Si}O_{2}\left(\text{clay}\;\text{silica}\right)\to C-S-H$$

$$Ca^{2+}+2{\left(\text{OH}\right)}^{-}+Al_{2}O_{3}\left(\text{clay}\;\text{alumina}\right)\to C-A-H$$

Where, $C=\text{CaO},\;A=Al_{2}O_{3},\;\text{and}\;H=H_{2}O$

Calcite also forms when Ca(OH)_2_ reacts with atmospheric CO_2_. This reaction is called carbonation. Calcite is crystalline and can also contribute to an increase in strength [25]. Haas [26] recorded up to 10% calcite as a result of carbonation in lime treated soil samples.

$$Ca^{2+}+2{\left(\text{OH}\right)}^{-}+CO_{2}\to \text{CaC}O_{3}+H_{2}O$$

Engineering tests such as unconfined compressive strength (UCS) test and plasticity index (PI) test are good indicators by which to assess the effect of pozzolanic reactivity in lime-treated soils. Addition of hydrated lime to a clayey soil facilitates modification and stabilization [27]. Modification occurs when Ca^2+^ ions (from hydrated lime) are adsorbed to the clay surface, lowering plasticity and flocculating the clay colloids [28]. The soil becomes friable and granular, making it easier to compact due to the reduced plasticity index [4]. Modification is followed by stabilization that occurs when the pH exceeds about 10.5 producing C-S-H as the reaction product (Equation 2). Therefore, UCS, pH and PI tests are used to validate pozzolanic reaction in the treated soils.

Analytical methods including using X-Ray diffraction (XRD) and differential thermogravimetry (DTA) are used extensively to detect C-S-H in hydrated cement pastes. The accuracy of these methods depends on sample preparation methods and the analysis of results [29, 30]. The same sample preparation for comparatively homogeneous hydrated cement pastes, for example, cannot be used for lime treated soils to detect C-S-H due to the heterogeneous nature and moisture sensitivity of soils. Therefore, we propose the following sample preparation steps for XRD and DTG analysis and a method to analyze the results for C-S-H from XRD and DTG.

## Material

The physical properties of the untreated soil samples S1 and S2 are shown in Table 1.

**Table 1:** Physical Properties.

Sample	Atterberg limits			Expansion potential	Gradation	
	Liquid Limit LL (%)	Plastic Limit PL (%)	Plasticity Index PI (%)		#200 (74 mm) passing (%)	Clay (%)
S1	75	29	46	High	97.4	57.1
S2	59	24	35	High	99.0	31.0

### Methods

**Fig. 1:**
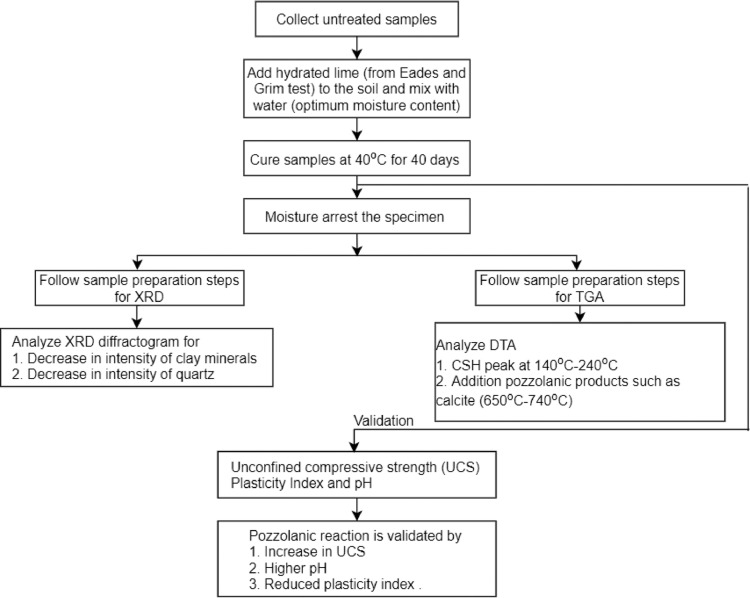
Methodology flowchart.

Fig. 1 summarizes the proposed steps for testing and validating pozzolanic reactions in lime-treated soils. The proposed method is applicable for both laboratory prepared lime-treated soil sample and field-treated samples.

### Engineering properties

A design hydrated lime dosage of 6% was determined on the basis of a threshold pH of 12.45 at 25°C for both soils evaluated in this study following ASTMD 6726-19 [31]. The treated sample were mellowed for 24 hours and compacted at optimum moisture content. The compacted samples were cured in a temperature-controlled chamber maintained at 40°C for 14 days. Unconfined compressive strength, pH and PI of the treated and untreated soil was determined in accordance to ASTM D2166 [32], ASTM D4872 [33], and ASTM D4318 [34], respectively. Three replicate samples were used for the unconfined compressive strength test.

### XRD sample preparation

The sample preparation method for untreated and lime-treated samples is as follows:

1. Recover a 5 g representative sample from the both the treated and untreated specimen.
2. Air-dry the sample for 72 hours.
3. Grind the representative treated and untreated sample with a mortar and pestle until the ground material passes the No. 325 (0.44 mm) sieve. If the samples stick to the sides of the mortar and pestle during the grinding process, making it difficult to grind, extend the air-dry period by 24 hours.

Addition of lime agglomerates clay sized fractions present in the soil and affects the XRD analysis. Therefore, the grinding process is important as it breaks down the agglomerated soil particles.

1. Back load onto the XRD sample holder. Back loading reduces the chances of preferred orientation due to stacking of clay minerals.
2. Run the XRD from 5^0^ to 70^0^ 2θ with the suggested parameters in Table 2.

**Table 2:** XRD instrument and scan parameters.

Data Collection properties		Settings
Sample	Sample dimension	26 mm diameter
	Spinning speed	15 rpm
	Sample type	Powder
	Sample pre-treatment	Grinding

Scan parameters	Sample loading	Back loading
	Angular range	5^0^– 70^0^ 2θ
	Scan rate	0.7^0^ 2θ/min
	Total measurement time	45.5 min

### DTA sample preparation

The sample preparation steps used for the DTA analysis are as follows:

1. Follow steps 1 to 4 for XRD sample preparation as previously described.
2. Moisture equilibrate both treated and untreated samples in a desiccator with CaSO_4_ as the desiccant for 14 days.
3. Test moisture equilibrated samples immediately at the following settings.

Table 3

**Table 3:** DTA instrument parameters.

Data Collection properties		Settings
Sample	Sample type	Powder
	Sample pre-treatment	Grinding
	Sample holder material	Alumina
	Minimum sample weight	50 mg

Test parameters	Temperature range	40°C to 1000^0^C
	Heat rate	20^0^C/min
	Purging gas	N_2_ (Nitrogen)
	Gas flow rate	30 mL/min
	Total measurement	90 min

## Results and discussion

### X-ray diffraction

**Fig. 2:**
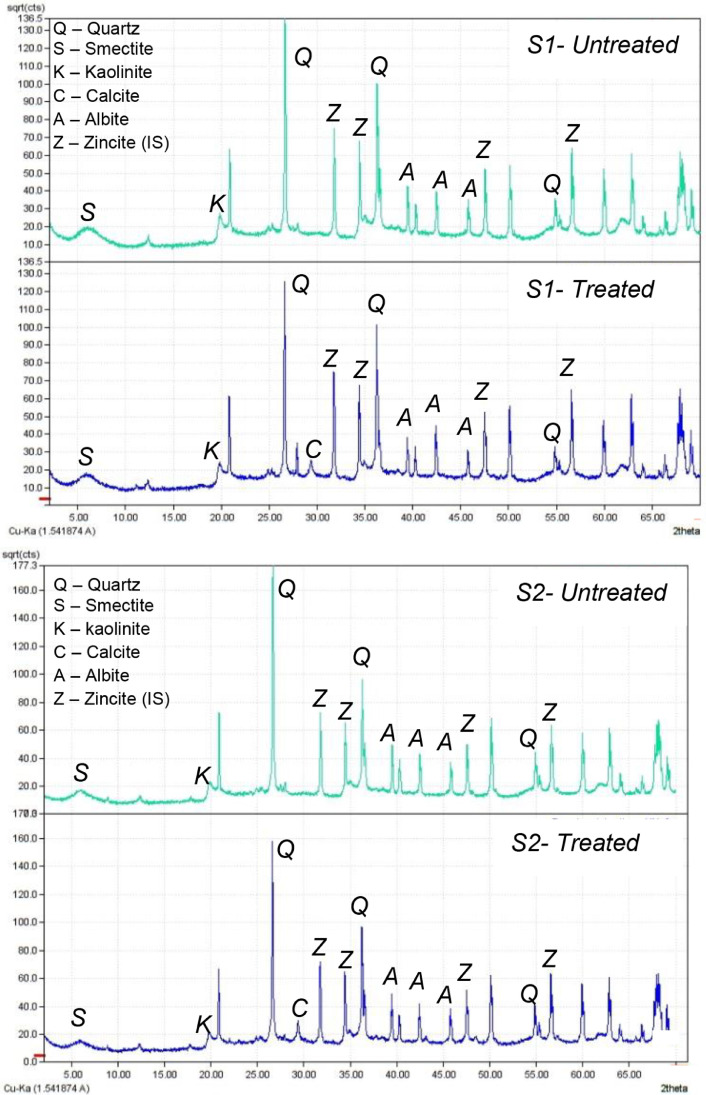
X-Ray diffractogram of samples S1 (top) and S2 (bottom).

Match! software was used to analyse the XRD data [35]. Fig. 2 shows the diffractograms for the treated and untreated specimens. Soil mineral structure files to identify soil minerals were obtained from crystallography open database [36]. XRD peaks indicated the presence of soil minerals smectite, kaolinite, albite, quartz, and calcite. It is evident from Fig. 2 that both treated samples, S1 and S2, showed two distinct differences in peak intensity when compared with the untreated samples. We recorded a reduction in peak intensity of quartz at 2θ 26.2° for the treated sample. The peak intensity reduced from 18,509 counts for the untreated samples to 15,652 counts (15.4% reduction) for treated sample S1. The peak intensity for the treated sample S2 reduced from 31,216 counts to 24,721 counts (20.8% reduction). This can be attributed to the partial destruction of silicate mineral structure including quartz due to high pH in the lime treated soils. Dhar and Hussain [37], and Norrish and Taylor [38] also observed a reduction in intensity for quartz. A reduction in the peak intensity of smectite and kaolinite was also observed. The partial dissolution of clay minerals is essential for pozzolanic reactions as indicated by Eades and Grim [39], and Bell [9]. Addition of hydrated lime increased the pH to between 10 and 12, which decreases the stability of kaolinite, smectite and quartz. Partial dissolution of quartz and other silicate mineral structures, smectite, and kaolinite [40] releases amorphous silica (SiO_2_ (am), equation 1) that can further react with Ca^2+^ (from hydrated lime) in the presence of H_2_O to form the pozzolanic product C-S-H. Furthermore, atmospheric CO_2_ can react with lime to produce calcite. The XRD diffractogram also shows the presence of calcite in the treated samples S1 and S2 (Fig. 2).

### Differential thermogravimteric analysis

**Fig. 3:**
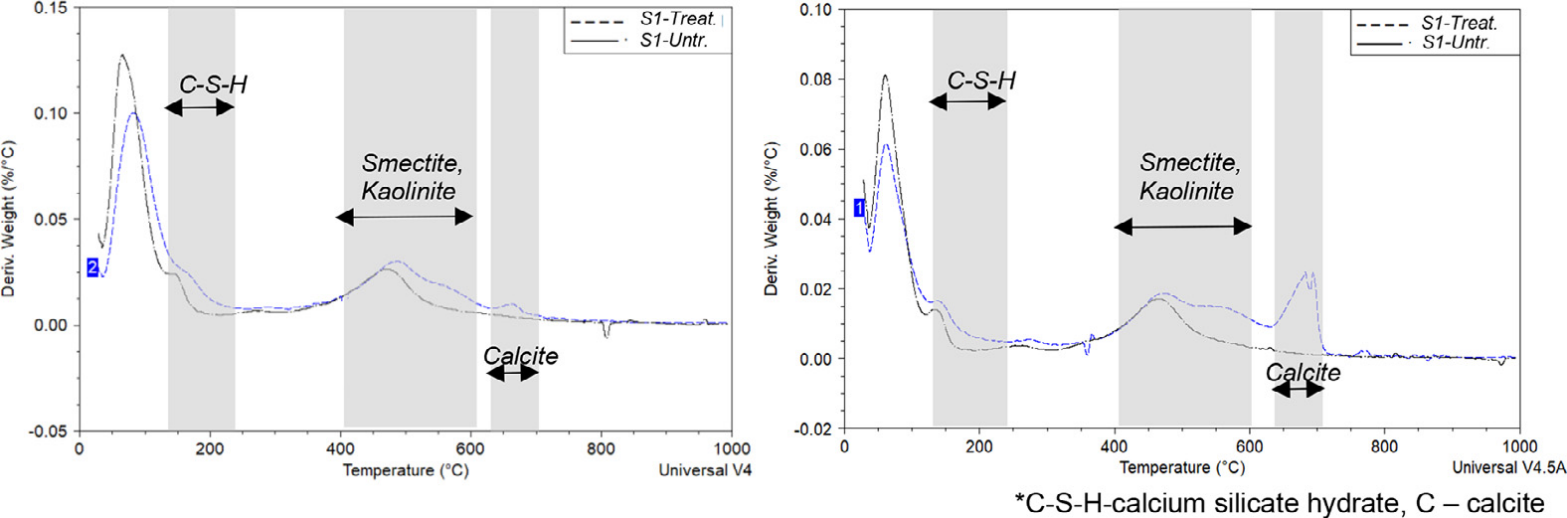
Differential thermogravimteric analysis plots of samples S1 (left) and S2 (right).

Differential thermogravimteric analysis (DTA) is a widely applied technique to measure hydration and carbonation by cement scientists. The DTA analysis results of the treated and untreated samples are shown in Fig. 3. In this study, DTA was used to identify pozzolanic products such as C-S-H. The temperature region from 0°C to 140°C can be attributed to moisture loss. Similar observations were made by Al-Mukhthar [13] for lime treated samples. In the treated sample, Ca^2+^ absorption on the net negative surfaces of smectite can affect moisture content. The difference in peak height between the treated and untreated samples at 100°C also indicated that the treated sample contained less moisture as compared to the untreated sample. Karen [30] showed that the temperature region for C-S-H occupies a wide area between 0°C to 300°C for hydrated cement pastes. The temperature region between 140°C and 240°C has been used to identify the formation of C-S-H in the treated soil. The treated samples S1 and S2 show a higher peak in the 140°C to 240°C region when compared with the untreated sample indicating the presence of C-S-H. In addition, the calcite (C) peak at 640°C to 700°C indicates carbonation reactions. Peaks from soil minerals such as smectite and kaolinite are seen in the region 400°C to 600°C [41, 42].

### Engineering properties

The maximum dry density and optimum moisture content (OMC) of the treated and untreated samples are shown in Table 4. After lime treatment, the OMC decreased for S1 and increased for S2. This may due to lower clay sized fractions in S2. In addition, both the treated samples recorded a reduction in maximum dry density.

**Table 4:** Compaction properties of the treated and untreated samples S1 and S2.

Sample	Untreated		Treated (6% lime)	
	Maximum dry density (kg/m^3^)	Optimum water content (%)	Maximum dry density (kg/m^3^)	Optimum water content (%)
S1	1550.5	22.2	1504.1	19.4
S2	1782.8	15.5	1654.7	17.9

**Fig. 4:**
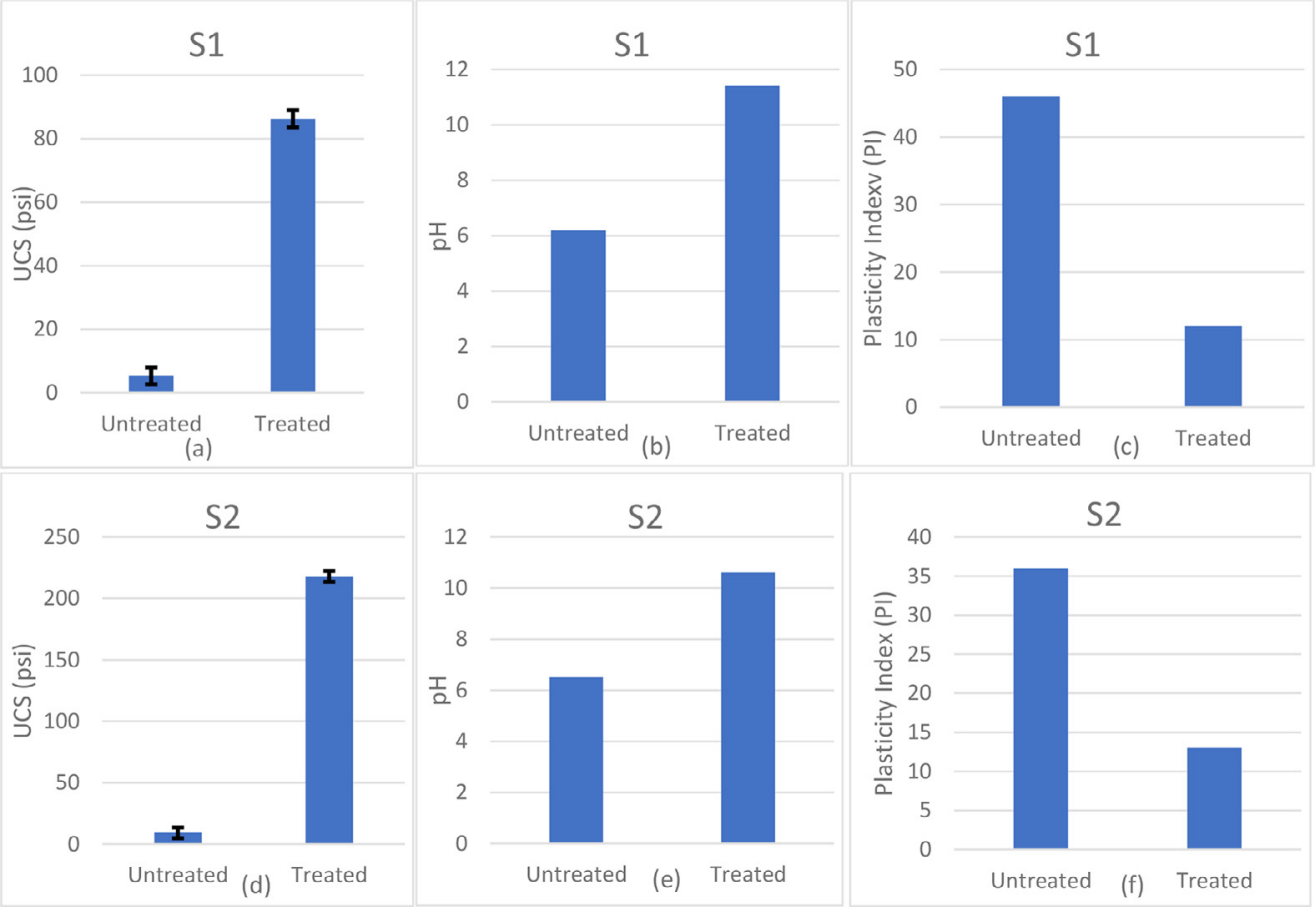
UCS, pH ad PI values of treated and untreated samples S1 and S2.

Fig. 4 shows UCS, pH and PI test results for the untreated and treated soils S1 and S2. Addition of hydrated lime increases the mean strength due to the stabilization reaction from 10 psi to 90 psi for soils S1 and from 10 psi to 220 psi for soil S2. In addition, an increase in pH from 6.2 to 10.8 for S1 and 6.2 to 10.5 for S2 also indicates potential for further pozzolanic reaction. A significant decrease in PI from 45 to 12 for S1 and from 36 to 12 for S2 shows the effect of modification of the soil due in part to pozzolanic reaction and calcium ion adsorption. Mineralogical analysis using DTA and XRD can be used to determine the magnitude of the pozzolanic product, which is considered a more durable reaction, and, therefore, the more resistant to reversal under certain environments.

## Conclusions

1. It is of key importance to be able to determine the degree of pozzolanic reaction developed in a lime-treated soil in order to assess the durability of the reactions responsible for changing the properties of the treated soil. The DTA and XRD analysis methods described herein are effective and efficient tools for assessing the degree of pozzolanic product.
2. DTA can qualitatively evaluate C-S-H precipitation.
3. The XRD diffractogram of the treated samples can quantify the level of reactivity and dissolution of quartz in the treated samples.
4. DTA and XRD in combination with traditional pH, PI and UCS tests combine to provide an effective and efficient protocol for assessing the quality and durability of lime-treated soils.
